# Systematic Functional Analysis of *PINK1* and *PRKN* Coding Variants

**DOI:** 10.3390/cells11152426

**Published:** 2022-08-05

**Authors:** Benjamin J. Broadway, Paige K. Boneski, Jenny M. Bredenberg, Ana Kolicheski, Xu Hou, Alexandra I. Soto-Beasley, Owen A. Ross, Wolfdieter Springer, Fabienne C. Fiesel

**Affiliations:** 1 Department of Neuroscience, Mayo Clinic, Jacksonville, FL 32224, USA; 2 Neuroscience PhD Program, Mayo Graduate School of Biomedical Sciences, Mayo Clinic, Jacksonville, FL 32224, USA; 3 Department of Clinical Genomics, Mayo Clinic, Jacksonville, FL 32224, USA

**Keywords:** mitophagy, PINK1, PRKN, Parkin, Parkinson, PD genes, ubiquitin

## Abstract

Loss of either PINK1 or PRKN causes an early onset Parkinson’s disease (PD) phenotype. Functionally, PINK1 and PRKN work together to mediate stress-activated mitochondrial quality control. Upon mitochondrial damage, PINK1, a ubiquitin kinase and PRKN, a ubiquitin ligase, decorate damaged organelles with phosphorylated ubiquitin for sequestration and degradation in lysosomes, a process known as mitophagy. While several genetic mutations are established to result in loss of mitophagy function, many others have not been extensively characterized and are of unknown significance. Here, we analyzed a set of twenty variants, ten in each gene, focusing on understudied variants mostly from the Parkinson’s progressive marker initiative, with sensitive assays to define potential functional deficits. Our results nominate specific rare genetic *PINK1* and *PRKN* variants that cause loss of enzymatic function in line with a potential causative role for PD. Additionally, we identify several variants with intermediate phenotypes and follow up on two of them by gene editing midbrain-derived neuronal precursor cells. Thereof derived isogenic neurons show a stability defect of the rare *PINK1* D525N mutation, while the common *PINK1* Q115L substitution results in reduced kinase activity. Our strategy to analyze variants with sensitive functional readouts will help aid diagnostics and disease treatment in line with current genomic and therapeutic advances.

## 1. Introduction

Parkinson’s disease (PD) is the second most common neurodegenerative disorder affecting 1% of the population over 60 years of age. Early onset PD, defined as onset before 40 years of age, is linked to the loss of *PINK1* or *PRKN* gene function in 1% or 5% of cases, respectively [1]. While some disease-linked single nucleotide variants (SNVs) have been shown to result in complete loss of function, the majority have not been tested yet and remain as variants of unknown significance. About 12 years ago, it was discovered that the ubiquitously expressed PINK1 and PRKN proteins act together in a common functional pathway to detect and dispose of damaged mitochondria via autophagy [2,3,4,5]. By virtue of many basic research studies that followed this seminal discovery, we have gained vast knowledge about the mechanistic details of how PINK1, the only known ubiquitin kinase, and PRKN, a ring between ring type E3 ubiquitin ligase, work in tandem to facilitate mitochondrial quality control. Functional assays to monitor different steps of the pathway were developed, however most studies, if not all, have used PINK1 or PRKN overexpression. Hence, there is a need to interrogate genetic *PINK1* or *PRKN* variants under endogenous expression. Harnessing post-translational modifications, PINK1 and PRKN generate phosphorylated ubiquitin that labels damaged mitochondria and mitochondrial proteins for degradation [6]. This tag promotes the recognition of damaged mitochondria by the autophagy system and facilitate the engulfment in autophagosomes to deliver damaged mitochondria to the lysosome where they are cleared [7].

Upon mitochondrial depolarization, the PINK1 kinase accumulates in the outer mitochondrial membrane. Its auto-phosphorylation further stabilizes PINK1 and enables the phosphorylation of ubiquitin [8,9,10,11,12,13]. Serine 65 phosphorylated ubiquitin (pS65-Ub) allosterically activates PRKN and recruits cytosolically located PRKN to the mitochondria [14,15,16,17]. Once recruited, PRKN is also directly phosphorylated by PINK1 in the ubiquitin like domain at its own conserved serine 65 residue [18,19,20,21]. In a feed-forward mechanism more PRKN molecules bring more ubiquitin to damaged mitochondria, and these serve as additional substrates for PINK1, which then activate and recruit more PRKN. As PRKN plays a critical role for pS65-Ub amplification, readouts of pS65-Ub can be used as a surrogate for both, PINK1 and PRKN activity [22].

Herein, we systematically analyzed a total of twenty variants in *PINK1* and *PRKN*, mostly identified in subjects enrolled in the Parkinson’s progression marker initiative (PPMI) [23]. Our selection included both rare and common SNVs (defined as <1%> gnomAD minor allele frequency). To assess variants for pathogenicity, we determined PINK1 and PRKN functionality based on the level of pS65-Ub alone, or in combination with their translocation to damaged mitochondria. Effects of two *PINK1* SNVs were further modeled in gene-edited midbrain-derived neurons under endogenous conditions. Our functional analysis of *PINK1* and *PRKN* SNVs will help to stratify patients in order to aid future disease treatments.

## 2. Materials and Methods

### 2.1. Variant Selection

*PRKN* and *PINK1* variants were identified across publicly available datasets (including PPMI) and in-house series of familial PD patients who have been screened for genes of interest. Variants were selected based on those which had not been extensively functionally characterized in previous literature and such included mostly benign or variants of unknown significance.

### 2.2. Cloning and Mutagenesis

*PRKN* variants were cloned by site-directed mutagenesis into the pEGFP-C1 vector containing PRKN-WT [24]; *PINK1* variants were cloned into pcDNA-PINK1-V5/6×His [2]. Briefly, forward and reverse mutant DNA fragments were amplified with a PCR cycler using forward and reverse mutagenesis primers (IDT, Coralville, IA, USA), ExTaq (Takara, San Jose, CA, USA), and selected vector templates. Amplified DNA was separated on 1% agarose gels, excised, and purified using a QIA gel extraction kit (Qiagen, Hilden, Germany). Then, forward and reverse mutant fragments were amplified together using external primers and purified using the same procedure. Amplified mutant DNA fragments were digested with specific restrictions enzymes (Fermentas, ThermoFisher Scientific, Waltham, MA, USA) or NEB (Ipswich, MA, USA)) and ligated with the previously described human PINK1 and PRKN pCMV driven expression vectors that were cut with matched restrictions enzymes, and de-phosphorylated. Ligations were transformed and plated onto either Ampicillin or Kanamycin (Sigma Aldrich, St. Louis, MO, USA) containing agar plates. Single colonies were expanded, and DNA was extracted using plasmid maxi kit (Qiagen) and sequenced using BigDye Terminator v.3.1 and an ABI 3100 Genetic Analyzer (Applied Biosystems, Thermo Fisher Scientific). Sequences were aligned and analyzed using SnapGene software (version 6.0.5, GSL Biotech, San Diego, CA, USA).

### 2.3. Cell Culture, Transfection, and Treatments

Human HeLa cells obtained from the ATCC (American Type Culture Collection) and human embryonic kidney cells 293E (HEK293E, Invitrogen, Thermo Fisher Scientific) were maintained in DMEM (Gibco, Thermo Fisher Scientific) plus 10% FBS (Neuromics, Edina, MN, USA) at 37 °C under humidified conditions and 5% CO_2_. Gene-edited PINK1 KO HEK293E have been described before [22]. mtKeima expressing cells were generated using lentivirus. Infected cells were sorted for neutral Keima fluorescence twice at 440 nm using a BD FACS Aria cytometer (BD Bioscience, Franklin Lakes, NJ, USA). Presorted mtKeima positive cells were seeded as single clones by limiting dilution. A clone with good signal-to-noise ratio of neutral Keima fluorescence cells was chosen and expanded. Midbrain-derived neuronal precursor cells ReNcell VM (Millipore, Burlington, MA, USA) were cultured on growth-factor reduced Matrigel (Corning, Corning, NY, USA) coated dishes in DMEM-F12 (Thermo Fisher Scientific) containing 1× B27 (Thermo Fisher Scientific), 5 U/mL Heparin (Sigma-Aldrich), and 50 µg/mL gentamicin (Thermo Fisher Scientific) as well as 20 ng/mL EGF and FGF (Peprotech, Cranbury, NJ, USA). Differentiation of ReN cells was performed by substituting FGF and EGF with 1 mM Dibutyryl-cAMP (Invivochem, Liberyville, IL, USA) and 2 ng/mL GDNF (Peprotech) for at least ten days [25].

Confluent HeLa or HeLa mtKeima cells were transiently transfected using 3 µL Lipofectamine 2000 (Thermo Fisher Scientific) and 1 µg of EGFP-PRKN cDNA following the manufacturers protocol. After 24 h HeLa cells expressing EGFP-PRKN WT or the EGFP-PRKN variants were treated with 20 µM CCCP (Sigma-Aldrich) for indicated time periods. HEK293E PINK1 KO cells were transiently transfected with Lipofectamine 2000 using 100–500 ng of PINK1-V5 WT or PINK1-V5 mutant variants per 1 million cells as an attempt to emulate low expressing endogenous conditions as described in [26].

### 2.4. SDS-PAGE and PhosTag

Cells were lysed in RIPA buffer (50 mM Tris-Cl pH 8.0, 150 mM NaCl, 1% NP-40, 0.5% deoxycholate, 0.1% SDS) supplemented with complete protease and PhosSTOP phosphatase inhibitors (Roche Applied Science, Penzberg, Germany). Protein concentration was determined using bichinoic acid (Thermo Fisher Scientific) and lysates subjected to SDS PAGE using 8–16% Tris-Glycine gels (Thermo Fisher Scientific). Proteins were transferred onto PVDF membranes (Millipore), blocked in 5% skim milk in TBST and probed with primary antibodies overnight at 4 °C. The next day, membranes were incubated with HRP-conjugated secondary antibodies (1:10,000; Jackson Immunoresearch Laboratories, West Grove, PA, USA). Images were acquired using Immobilon Western Chemiluminescent HRP Substrate (Millipore) on autoradiography X-ray films (Genesee Scientific, El Cajon, CA, USA) or using a chemidoc imaging system (Bio-Rad Laboratories, Hercules, CA, USA). PINK1 auto-phosphorylation was determined using freshly prepared cell lysates with EDTA-free complete inhibitors. Samples were separated on 6% Tris-Glycine gels that contained 100 µM Phos-Tag acrylamide (Wako Chemicals, Richmond, VA, USA) and 50 µM MnCl_2_. Before transfer onto nitrocellulose membranes (Bio-Rad Laboratories), gels were washed for 20 min twice in transfer buffer containing 1 mM EDTA and 0.01% of SDS and twice in the absence of EDTA. All steps downstream resembled those previously described.

### 2.5. Antibodies

PINK1 expression was determined using anti-V5 (1:1000 Abcam ab9116) and anti-PINK1 (1:5000 CST D8G3 or 1:2000 Biolegend DU46.1-1). Membranes were probed with anti-pS65-Ub (1:1000 CST 37642 or 1:20,000 CST E2J6T) as a readout for substrate phosphorylation. Anti-PRKN (1:5000 Millipore PRK8) was used to determine PRKN expression levels in ReN cells by Western blot and MFN2 (1:2000 Abcam ab56889) as a substrate of PRKN. GAPDH (1:100,000 Meridian Life Sciences H86504M) and Vinculin antibodies (1:200,000 Sigma V9131) were probed for loading controls.

### 2.6. Densitometry and Statistical Analysis

Densitometry analysis of Western blot was performed by using Image Studio Lite Ver 5.2. GraphPad Prism version 9 was used for data visualization and analysis. All quantitative results are expressed as mean ± SEM from at least three independent experiments. Statistical comparisons were performed using either a one-way or two-way ANOVA with Dunnett’s post hoc test for more than two groups or two-way ANOVA with Sidak’s post hoc comparison for two groups, as indicated in the figure legends (* *p* < 0.05, ** *p* < 0.005, *** *p* < 0.0005).

### 2.7. Immunofluorescence Staining, Microscopy, and High Content Imaging

For immunofluorescence staining, HeLa cells were plated onto poly-d-lysine (Sigma Aldrich) coated cover slips in a 24-well plate and then transfected as previously described. For high content imaging, cells were transfected in 6-well plates, and after 24 h cells were detached, counted, and plated in 96-well imaging plates (Falcon, Corning, NY, USA). For both, cells were allowed to adhere for 24h before being treated with 20 µM CCCP and fixed in 4% PFA. Isogenic neurons were differentiated on Matrigel-coated coverslips and also fixed with 4% PFA. Cells were permeabilized with 1% Triton-X100 in PBS before being incubated with primary antibodies (anti-HSP60 [Arigo ARG10757], anti-pS65-Ub [CST 37642S], anti-TOM20 [Proteintech group 11802-1-AP], anti-LAMP2 [Developmental Studies Hybridoma Bank H4B4c], MAP2 [Abcam ab5392]) and secondary AlexaFluor antibodies (Thermo Fisher Scientific). Nuclei were stained with Hoechst 33342 (Thermo Fisher Scientific). For immunofluorescence microscopy, cover slips were mounted and imaged with a 63× objective using a Zeiss AxioObserver with Apotome for confocal imaging. High content image acquisition and analysis was performed as described in [27]. To exclude untransfected as well as highly overexpressing cells, only cells with a GFP signal 40% above the background and less than 400% of the average signal were considered for analysis.

### 2.8. Flow Cytometry

HeLa mtKeima cells transfected with either EGFP-PRKN WT or the EGFP-PRKN variants were either left untreated or treated with 20 µM CCCP for 4 or 8 h. Cells were lifted from 6-well plates using 0.05% Trypsin with EDTA (Invitrogen), washed in PBS (Gibco) and finally re-suspended in FACS buffer (10 mM HEPES in 1× HBSS containing 2% FBS). Cells were analyzed for the Keima fluorescence intensity at both excitations 420 nm and 561 nm using flow cytometry (Attune NxT). In order to exclude un-transfected cells, a population of 40,000 EGFP-positive cells were selected for analysis. To view PRKN mediated mitophagy, for each variant the background was subtracted, and the adjusted raw values were then normalized between WT (set to 1) and C431S (set to 0).

### 2.9. CRISPR/Cas9 Gene-Editing

We used CRISPR/Cas9 to introduce single nucleotide exchanges into ReN cell VM. Guide sequences (Q115L: CCTCATCGAGGAAAAACAGG, D525N: CTGAAGTTAGACAAGATGGT) were cloned into MLM3636 (Addgene #43860) and transfected together with a construct expressing GFP-tagged Cas9 (Addgene #44719) and a symmetric 200 bp single stranded oligo using a Nucleofector P3 kit (Lonza, Basel, Switzerland). 48–72 h after transfection, GFP-positive cells (usually around 30–50%) were purified by flow cytometry and then seeded as single clones by limited dilution after another 48h. Clone screening was performed after wells had visible colonies. DNA was extracted and analyzed by PCR and restriction digest followed by Sanger sequencing (Genewiz, Azenta Life Sciences, South Plainfield, NJ, USA). Sequences were analyzed using Snapgene software. Off-target edits were excluded by Sanger sequencing of the top five candidate regions as identified by the Benchling biology software (2021, www.benchling.com).

## 3. Results

### 3.1. Common PRKN Variants Do Not Show Discernible Functional Deficits

**Figure 1:**
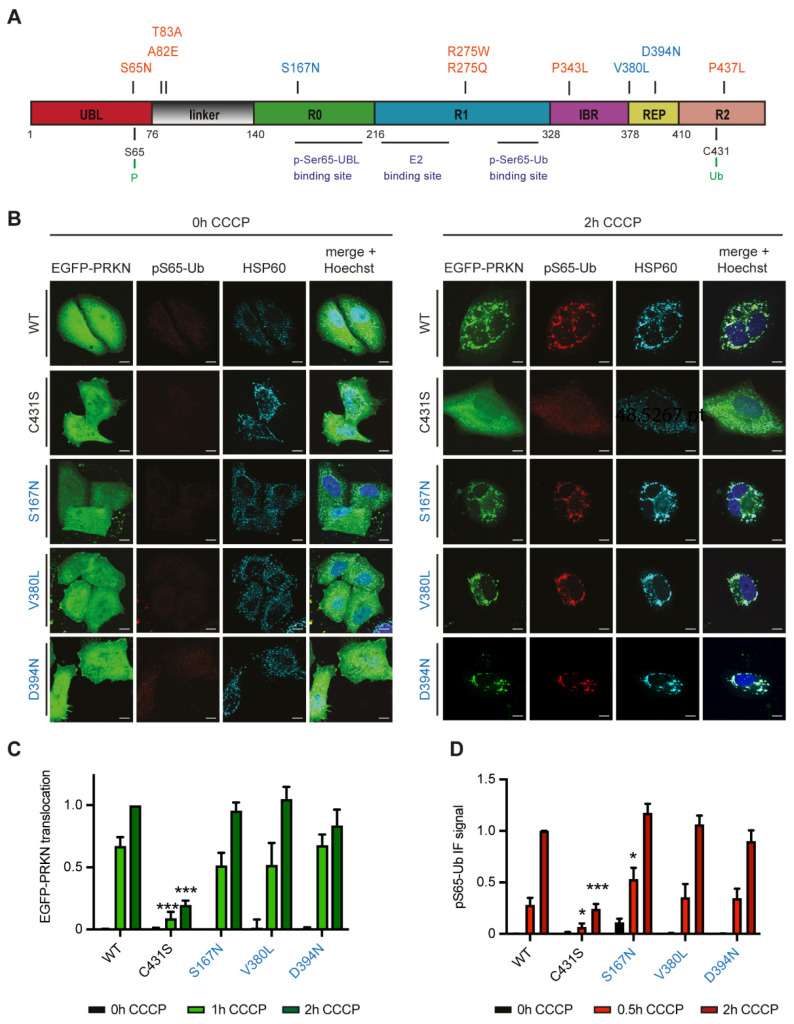
Functional analysis of frequent PRKN variants. (**A**) Organization of PRKN protein with investigated SNVs. Protein domains are drawn to scale and color-coded (red: ubiquitin like domain, grey: linker region, green: RING0/unique PRKN domain, blue: RING1, Purple: in-between-RING domain, yellow: repressor element of PRKN, pink: RING2). Residues Ser65 and Cys431 as well as regions for intramolecular and intermolecular interactions, which are important for PRKN activation and enzymatic function are highlighted below. Rare variants are indicated in orange (<1% MAF), and frequent variants in blue. (**B**–**D**) HeLa cells were transfected with frequent PRKN variants that were N-terminally EGFP-tagged and either left untreated or treated with CCCP, as indicated. Cells were fixed and stained with antibodies against pS65-Ub and HSP60 as mitochondrial marker and nuclei counterstained with Hoechst 33342. Representative images are shown (**B**). Upon treatment with CCCP, EGFP-PRKN WT but not C431S co-localized with the mitochondrial marker (HSP60, cyan). PRKN co-localization with mitochondria coincided with a visible increase in pS65-Ub at the mitochondria. Scale bars correspond to 10 µm. Cells were imaged using a HCI microscope and analyzed for PRKN translocation (**C**) or pS65-UB signal intensity (**D**). Data of each experiment was normalized to between 0 h and 2 h for GFP-PRKN WT, set to 0 and 1, respectively. Data is shown as the mean ± SEM of at least six independent experiments with four replicate wells per condition. Statistical analysis was performed using two-way ANOVA and Dunnett’s multiple comparisons test (* *p* < 0.05, *** *p* < 0.0005).

We nominated a total of ten *PRKN* variants for a detailed functional analysis containing seven rare variants found either in the PPMI or in a familial PD cohort and three common variants with a gnomAD minor allele frequency > 1% (Figure 1A). Selected mutations were cloned into PRKN cDNA constructs with an N-terminal EGFP tag and transiently transfected into HeLa cells that lack endogenous PRKN expression [28,29]. We expressed EGFP-PRKN wild-type (WT) and a catalytically inactive EGFP-PRKN C431S mutant [30] as positive and negative control, respectively. For PRKN WT and common PRKN variants, CCCP treatment led to the redistribution of EGFP-PRKN from the cytoplasm to mitochondria, while PRKN C431S remained cytoplasmic (Figure 1B). PRKN translocation to mitochondria was accompanied by the appearance of pS65-Ub fluorescence intensity. We further quantified the translocation of PRKN and the signal intensity of pS65-Ub by High Content Imaging (HCI) (Figure 1C,D) while excluding non-transfected, EGFP-negative cells from this analysis. pS65-Ub levels were normalized to the matching EGFP signal for each cell to account for expression differences. Coherent with the manual observation, none of the common PRKN SNVs showed a significant delay in PRKN translocation or major differences in pS65-Ub signal intensity compared to WT using automated HCI. For PRKN S167N, there was a slight increase of pS65-Ub at the 30 min time point but not after 2 h of CCCP treatment, indicating a transient nature of this effect. In both HCI readouts, PRKN translocation to mitochondria and pS65-Ub levels, the ligase-dead PRKN C431S variant showed a consistent reduction compared to PRKN WT, as expected.

### 3.2. Specific Rare PRKN Variants Display Reduced Translocation and Enzymatic Function

**Figure 2:**
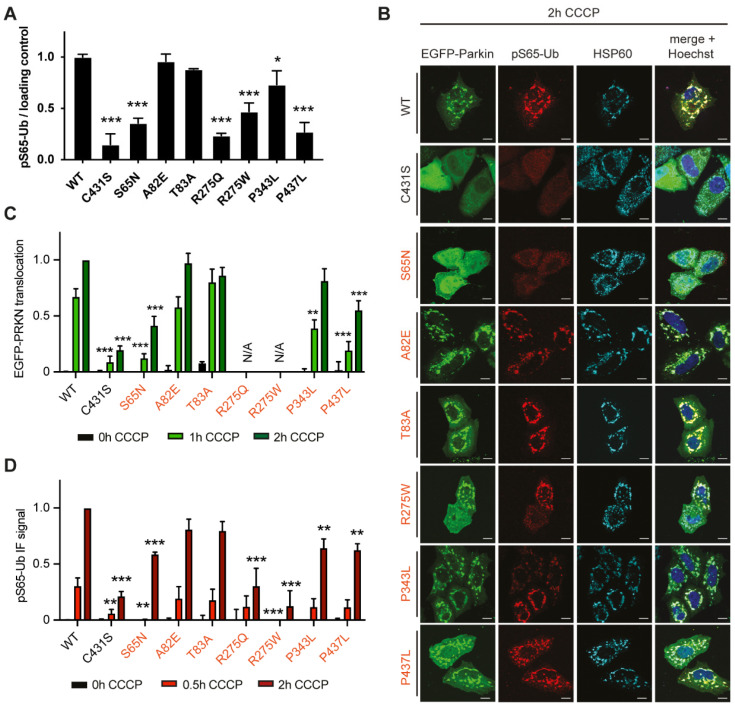
Functional analysis of rare PRKN variants. HeLa cells were transfected with either EGFP-PRKN WT, the catalytically inactive C431S, or rare PRKN variants and treated with CCCP, as indicated. (**A**) Western blots were prepared and immunoblot pS65-Ub levels were quantified and normalized to loading control. Data is shown as the mean ± SEM from at least three independent experiments. Statistical analysis was performed using one-way ANOVA with Dunnett’s multiple comparison test (* *p* < 0.05, *** *p* < 0.0005). (**B**) Cells were fixed and stained with antibodies against pS65-Ub (red) and HSP60 (cyan) and nuclei counterstained with Hoechst 33342 (blue). Scale bars correspond to 10 µm. (**C**,**D**) Cells prepared as in B were imaged using HCI and analyzed for PRKN translocation (**C**) or pS65-UB signal intensity (**D**). The data of each experiment was normalized between 0 h and 2 h for EGFP-PRKN WT, set to 0 and 1, respectively. Data is shown as the mean ± SEM of at least three independent experiments with four replicate wells per condition. Statistical analysis was performed using two-way ANOVA with Dunnett’s post hoc test to correct for multiple testing (** *p* < 0.005, *** *p* < 0.0005).

We next analyzed the rare *PRKN* variants (Table 1). HeLa cells were transiently transfected with EGFP-tagged PRKN variants and treated with CCCP for 2 h in analogy to the above. Western blots were prepared and pS65-Ub signals quantified and normalized to the loading control (Figure 2A). Compared to WT, several PRKN variants showed a highly significant reduction of pS65-Ub. This included the PRKN S65N variant, highlighting the importance of the PINK1 phosphorylation site in the UBL domain of PRKN (Figure 2A) [31]. pS65-Ub levels were also lower for PRKN R275W, which had previously been reported to disrupt pS65-Ub binding and destabilize the protein [32], impairing catalytic activity. Both the PRKN R275W and R275Q variants demonstrated significantly reduced pS65-Ub levels compared to PRKN WT. We also observed very low pS65-Ub levels for the PRKN P437L variant, which is located in the RING2 domain close to the catalytic C431 residue and amino acids C436, C441, C421 and C418 that coordinate a zinc ion and hence might lead to a conformational disruption of the active site. Another proline to leucine substitution, PRKN P343L is located in the IBR domain away from the active site and displayed a less drastic reduction of pS65-Ub, while PRKN A82E and T83A did not show any obvious reduction in pS65-Ub Western blot levels.

Immunostaining confirmed that the PRKN S65N and R275W variants found in the PPMI cohort reduced PRKN translocation and diminished pS65-Ub intensity (Figure 2B–D). We consistently observed reduced PRKN translocation also for the PRKN variants S65N and P437L by HCI quantification (Figure 2C). The two PRKN substitutions at position 275 demonstrated a large spread of values for the subcellular location of PRKN under basal conditions, possibly indicating aggregation [33], and were thus omitted from the analysis of PRKN translocation (Figure 2C). The quantification of pS65-Ub intensity per cell by HCI further supported significant reduction of function for PRKN S65N, R275Q, R275W, P343L and P437L (Figure 2D). The two PRKN variants in the linker region, A82E and T83A, displayed a similar enzymatic profile as PRKN WT in all assays (Figure 2A–D), indicating the E3 ubiquitin ligase may be able to tolerate these single nucleotide variants in the linker domain and still execute mitophagy.

### 3.3. Certain PRKN Variants Affect Mitochondrial Turnover Rates

**Figure 3:**
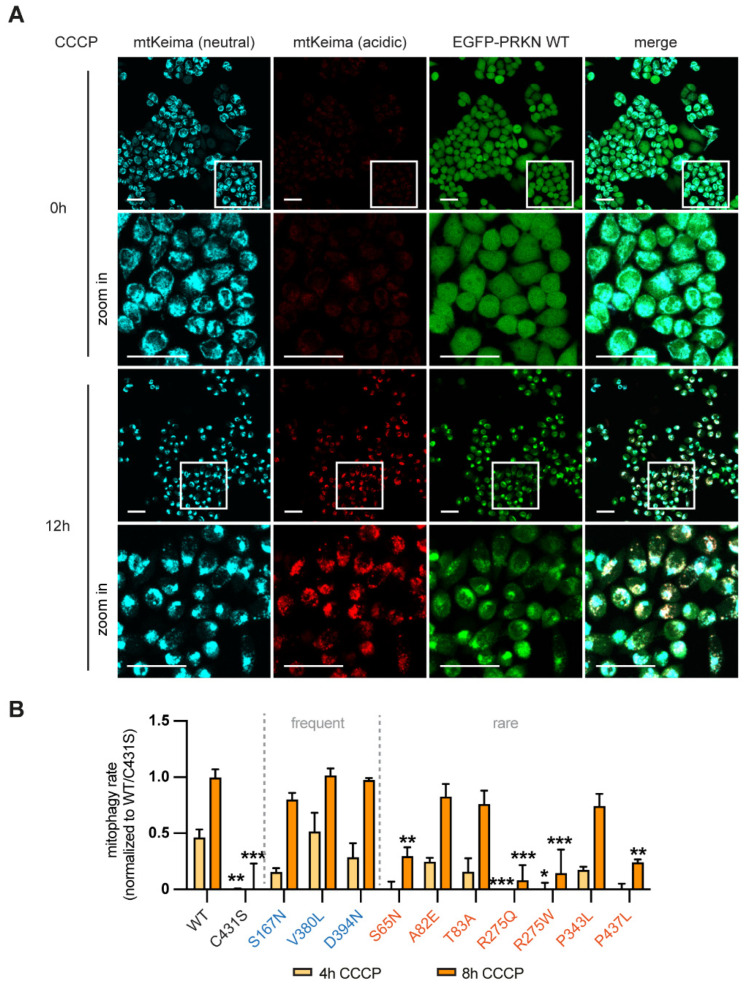
Analysis of mitochondrial flux in cells transfected with PRKN variants. (**A**) Representative images of HeLa cells stably expressing mtKeima and EGFP-PRKN WT with and without 12 h CCCP treatment. Images detail an increase of signal with 561 nm excitation (red, acidic mtKeima) and the presence of EGFP-PRKN clusters (green) at the mitochondria after CCCP treatments, while the signal obtained with 450 nm excitation (blue, neutral mtKeima) stays relatively consistent. Scale bars correspond to 50 µm. (**B**) HeLa cells stably expressing mtKeima were transiently transfected with EGFP-PRKN variants, treated for 4 or 8 h with CCCP and analyzed by flow cytometry. The average ratio of excitation at 561 nm: 450 nm was calculated from the signal heights of 40,000 SYTOX Red negative and EGFP-PRKN positive cells and normalized to WT and C431S. Data is shown as the mean ± SEM of at least three independent experiments. Statistical analysis was performed using two-way ANOVA with Dunnett’s post hoc test to correct for multiple testing (* *p* < 0.05, ** *p* < 0.005, *** *p* < 0.0005).

Mitophagy is a multi-stage process that results in the fusion of damaged mitochondria with lysosomes where they are degraded. This turnover can be assessed by measuring the ratio of differentially excited mitochondrially targeted Keima (mtKeima), a pH-dependent fluorescent reporter protein [34,35]. The signal of acidic mtKeima is robustly induced upon CCCP treatment in HeLa cells stably expressing EGFP-PRKN WT (Figure 3A). To test the effect of different genetic variants, we transiently transfected EGFP-tagged PRKN cDNAs into mtKeima cells and measured the ratio of acidic over the neutral mtKeima signal for each cell by flow cytometry. We restricted our analysis to live, transfected cells by gating only EGFP-positive and SYTOX red negative cells. The results were normalized to data obtained with cells overexpressing PRKN WT or C431S and expressed as percent mitophagy. No variant displayed unusual mitophagy rates at baseline (data not shown) and common PRKN variants did not show reduced mitophagy rates upon CCCP treatment (Figure 3B), consistent with above findings. However, PRKN variants that caused significantly reduced pS65-Ub levels (S65N, R275W, R275Q and P437L), also caused clear mitophagy deficits. Yet, the milder reduction of pS65-Ub levels that were seen with P343L by both Western blot and immunofluorescence did not translate into reduced mitophagy rates. Again, coherent with the readouts for mitophagy activation, the two PRKN SNVs mapped to the PRKN linker region, A82E and T83A, displayed mitochondrial degradation rates similar to PRKN WT (Figure 3B).

### 3.4. Functional Analysis of Common and Rare PINK1 Variants

**Figure 4:**
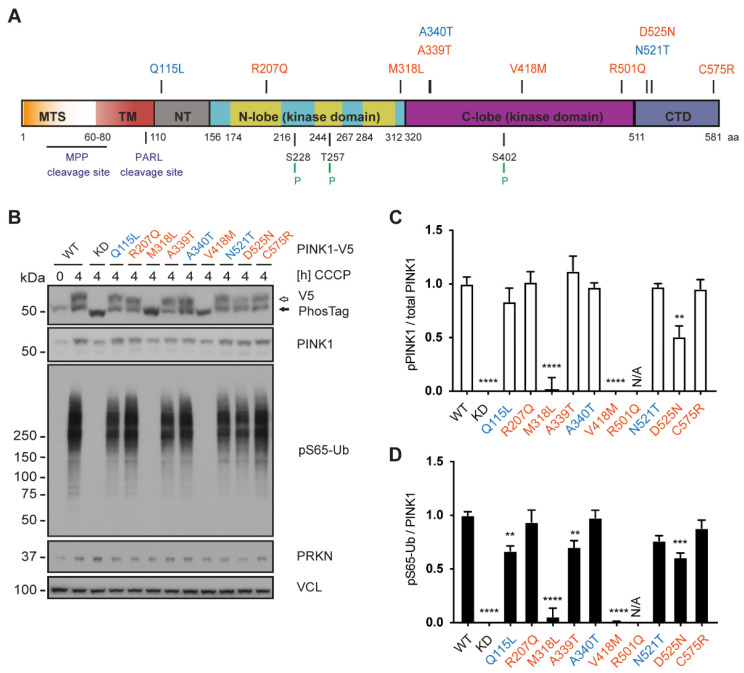
Functional analysis of genetic PINK1 variants. (**A**) Organization of PINK1 protein with the location of investigated SNVs. Protein domains are drawn to scale and color-coded (orange: mitochondrial targeting sequence, red: transmembrane domain, grey: N-terminal, blue/purple: N-lobe/C-lobe kinase domains with insertion loops highlighted in yellow, dark blue: C-terminal domain). Protease cleavage sites and auto-phosphorylation sites labelled below. Investigated PINK1 variants are listed at the top: rare variants in red, and frequent PINK1 SNVs in blue. (**B**) HEK293E PINK1 KO cells were transfected with either PINK1-V5/6xHis WT, kinase-dead (KD) or the genetic PINK1 variants and left untreated or treated with CCCP, as indicated. Standard and phostag electrophoresis were performed and Western blots prepared. Blots were probed with V5 and PINK1 antibodies to assess the levels of total and phosphorylated PINK1 kinase. pS65-Ub antibodies were used to determine the amount of PINK1 product. PRKN levels were unaffected by transfection of PINK1 variants. VCL was used as loading control. (**C**,**D**) Quantification of Western blots shows normalized values for phosphorylated PINK1 protein over total PINK1 protein (**C**) and pS65-Ub over total PINK1 (**D**) for each variant. Data from at least four independent experiments was normalized to WT and KD, set to 1 and 0, and is shown as mean ± SEM. As PINK1 R501Q was not stabilized upon CCCP, it was omitted from the quantification. Statistical analysis was performed using one-way ANOVA with Dunnett’s post hoc test to correct for multiple testing (** *p* < 0.005, *** *p* < 0.0005, **** *p* < 0.0001, N/A not applicable).

We also selected a total of ten *PINK1* variants for functional characterization, eight of which were found in the PPMI (Figure 4A). Among those were three common *PINK1* variants (Q115L, A340T, N521T) as well as seven rare SNVs with a gnomAD minor allele frequency < 1% (Table 2). All variants were first cloned into cDNA constructs with C-terminal V5/6xHis tag [2] for transfection into HEK293E cells where we had knocked out PINK1 by CRISPR/Cas9 to remove confounding endogenous PINK1 activity [22]. To observe and compare the stabilization of PINK1 SNVs upon mitochondrial damage, we first optimized a transfection protocol to obtain consistent low-level expression of mainly full-length PINK1, which represents the kinase-active form of PINK1 (Supplementary Figure S1A). Mitochondrial depolarization was then induced with CCCP, and protein lysates analyzed by Western blot. In contrast to PINK1 WT and all other variants, a Western blot band for PINK1 R501Q was not visible upon CCCP treatment even with long exposure times (see Supplementary Materials, Figure S1B) and consistently pS65-Ub levels were barely detectable for this mutant. Expression of R501Q could be observed upon treatment with MG132 [26], suggesting that PINK1 R501Q fails to stabilize upon CCCP. We further complemented regular gel electrophoresis with phostag gels for all other SNVs to assess PINK1 autophosphorylation. Phostag is a small molecule that retards phosphorylated species, resulting in a band-shift of phosphorylated proteins (Figure 4B). Two rare PINK1 variants, M318L and V418M, that were found in the PPMI displayed no PINK1 auto-phosphorylation, indicating a loss of kinase activity for both variants, while overall protein levels of PINK1 remained relatively unchanged for all analyzed variants (Figure 4C). Consistently, we observed minimal ubiquitin phosphorylation for both PINK1 M318L and V418M, while all other PINK1 variants that displayed auto-phosphorylation also showed some degree of ubiquitin phosphorylation. Quantification of several repeat experiments normalized to total PINK1 levels uncovered a small but significant decrease in auto- and substrate phosphorylation for PINK1 D525N as well as reduced substrate phosphorylation for the common Q115L and the rare A339T variant (Figure 4D). Other variants, including R207Q, A340T, N521T and C575R were indistinguishable from PINK1 WT with regard to both auto- and substrate phosphorylation.

### 3.5. Predicted versus Actual Deleteriousness of PINK1 and PRKN Variants

**Figure 5:**
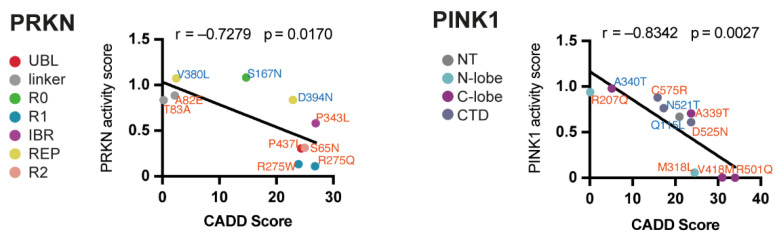
Correlation of functional activity with CADD score. Linear regression of composite activity scores for variants were based on the average of pS65-Ub Western blot, immunofluorescence signal and PRKN translocation (PRKN) or pS65-Ub Western blot levels (PINK1) show a significant and strong negative correlation with CADD scores for both, PRKN and PINK1.

We next performed correlations of the functional analysis results with the predicted deleteriousness for *PINK1* and *PRKN* variants. The CADD framework integrates multiple annotations into one metric and is considered one of the most accurate predictions for single nucleotide variants and deletions in the human genome [36,37]. A SNV with a CADD score of 20 or over 30 indicates that these SNVs belongs to the most deleterious 0.1% or 0.01% in the human genome, respectively. Using pS65-Ub Western blot signal as an activity score for PINK1 mutants and the average of pS65-Ub signal from Western blot, immunofluorescence, and PRKN translocation for PRKN mutants, respectively, the correlation with CADD revealed significant negative associations for variants in both *PINK1* and *PRKN* (Figure 5).

There were some instances where the CADD score and the functional activity were not congruent. For example, the PINK1 M318L substitution had a much more severe effect than predicted. Methionine 318 was previously identified as the gatekeeper residue of PINK1 that sits on the outer lip of the ATP pocket and guards access of ATP to the active site [38]. It seems that the importance of this specific site within the PINK1 structure for its activity was underestimated by the global CADD tool. In general, common substitutions in PINK1 and PRKN, shown in blue, had a relatively benign effect on their functional activity while the rare mutants either had very little or very dramatic defects. We further noticed a cluster of PINK1 protein changes with CADD scores between 20 and 30 including the common Q115L and rare D525N mutations and set out to investigate these further under endogenous conditions in disease relevant neurons.

### 3.6. Gene-Editing of PINK1 Variants in Midbrain-Derived Neuronal Precursor Cells

**Figure 6:**
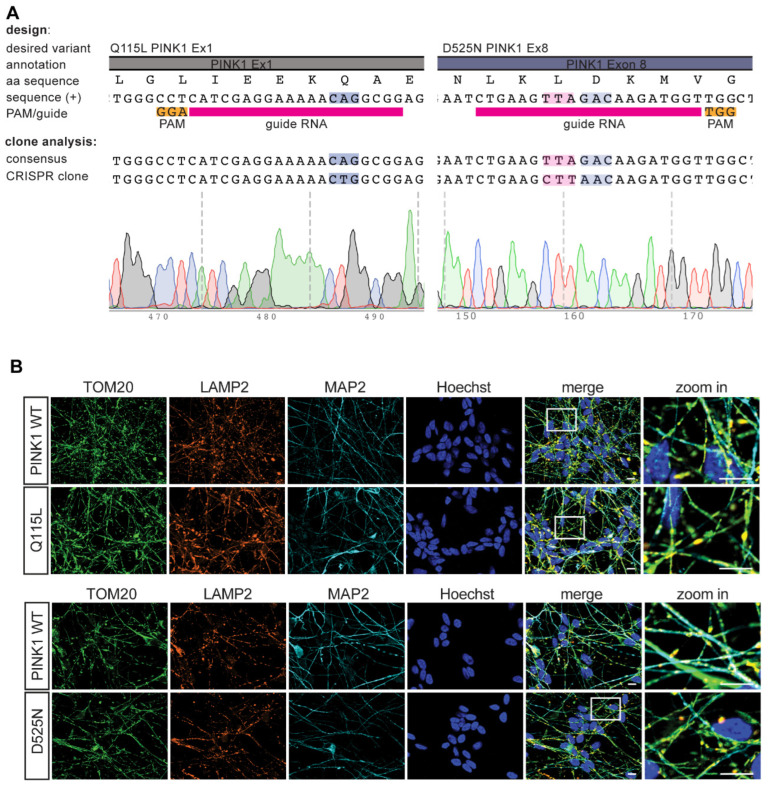
Gene-editing of PINK1 variants in mid-brain derived neuronal precursor cells. (**A**) Details for gene-editing of PINK1 WT to Q115L or D525N using CRISPR/Cas9 is shown from top to bottom with exon/intron structure, amino acid sequence, genomic sequence, guideRNA and PAM sequence, as well as results from Sanger sequencing of positive cell clones. Blue highlight indicates targeted triplet for intended amino acid change, pink highlight shows position of a silent mutation that was introduced to block re-editing and facilitate screening by restriction enzyme digest. (**B**) Mid-brain derived precursors were differentiated to neurons, fixed, stained and analyzed by immunofluorescence. Cells were stained with antibodies against TOM20 (mitochondria, green), LAMP2 (lysosomes, red) and MAP2 (neuronal marker, cyan). Nuclei were counterstained with Hoechst (blue). A zoom into the indicated region is shown to the right. Scale bars indicate 10 µm.

We introduced these two variants with intermediate functional into the endogenous *PINK1* locus by gene-editing. As a disease relevant model, we utilized the ReNcell VM line, human dopaminergic neuronal precursor cells with a stable diploid genome that can easily be differentiated into neurons. We designed guide sequences for targeting with CRISPR/Cas9 and 200 bp single stranded oligos as template for homologous recombination (Figure 6A). For D525N we added an additional silent mutation at the adjacent 5′ triplet (TTA [L] to CTT [L]) with similar codon usage to serve as blocking mutation and prevent re-editing of the WT allele [39] for the generation of heterozygous D525N clones. At the same time the silent blocking mutation would introduce a novel restriction enzyme (RE) site. Substitution of Q115L (CAG [Q] to CTG [L]) also led to the presence of a new RE site that could be used for pre-screening of clones.

We transfected ReNcell VM with guideRNA, a plasmid expressing GFP-tagged Cas9, and repair template by nucleofection, purified the cells by flow cytometry to sort GFP-positive cells, and seeded them into 96-well plates to grow as single clones. Screening was performed when wells had visibly grown colonies, after about 2 weeks. We screened about 150 clones per intended mutation but received only one clone for Q115L and eight clones for D525N (5 homo- and 3 heterozygous) without erroneous indels. We further excluded unwanted gene-editing at off-target sites and sequenced the top five loci each (data not shown) by Sanger sequencing.

We then differentiated the precursor cells into neurons and performed immunofluorescence staining (Figure 6B). The gene-edited cells with PINK1 Q115L or D525N variants looked indistinguishable from the isogenic parental cells with regard to gross neuronal morphology (neuronal marker MAP2) and the morphology of mitochondria (mitochondrial marker TOM20) and lysosomes (lysosomal marker LAMP2).

### 3.7. PINK1 D525N Leads to Destabilization of PINK1 Protein

**Figure 7:**
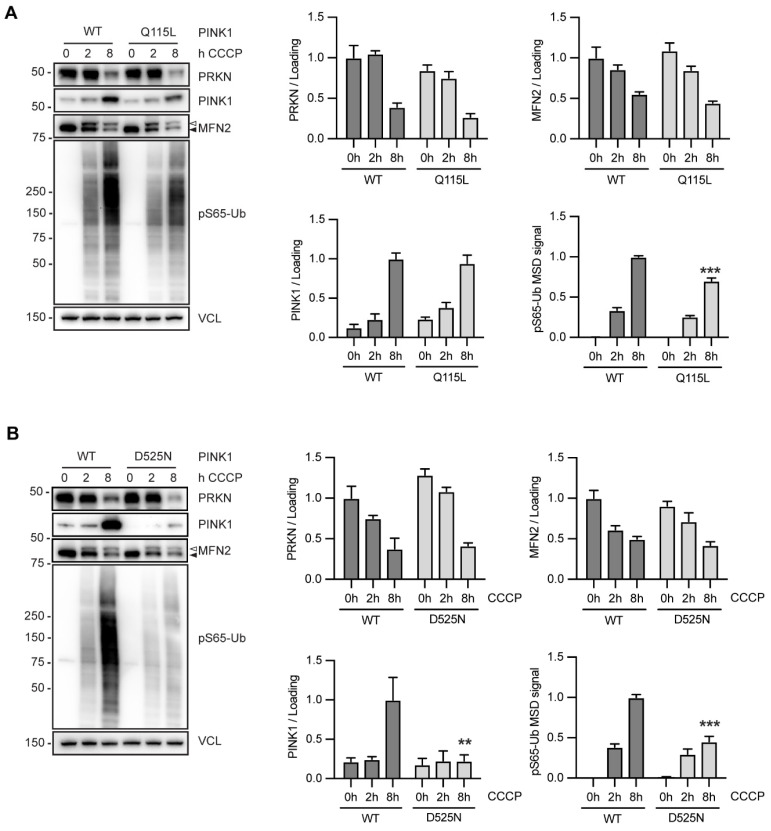
Analysis of PINK1 Q115L and PINK1 D525N in gene-edited neurons. (**A**,**B**) Differentiated neurons endogenously expressing either PINK1 WT or PINK1 Q115L (**A**) or WT or D525N (**B**) were treated with CCCP for 0, 2, or 8 h, respectively. Western blots were prepared and probed with antibodies for PRKN, PINK1, the PRKN substrate MFN2, pS65-Ub and VCL as loading control. pS65-Ub levels were also quantified by MSD sandwich ELISA. Data is shown as the mean ± SEM from three technical (**A**) or biological (**B**) replicate experiments. Statistical analysis was performed using two-way ANOVA with Sidak’s post hoc test to correct for multiple testing (** *p* < 0.005, *** *p* < 0.0005). Unmodified MFN2 is indicated by a closed, and ubiquitinated MFN2 by an open arrowhead. (**A**) PINK1, PRKN, and MFN2 levels remain unchanged in gene-edited Q115L, compared to WT, but pS65-Ub levels are significantly decreased. (**B**) PRKN, and MFN2 levels look similar between WT and gene-edited D525N, but PINK1 protein levels as well as pS65-Ub levels are significantly decreased in the mutant cells compared to controls.

To test the functional defect of PINK1 Q115L and D525N, derived neurons were treated with mitochondrial depolarizer in a time course experiment for 0, 2, and 8 h. PINK1 levels, PRKN levels, and MFN2 ubiquitination/turnover, indicated by the appearance of an upper MFN2 band and the disappearance of the lower MFN2 band in cells with PINK1 Q115L variant were unchanged compared to parental ReN neurons, although a small reduction in pS65-Ub level was observed. We quantified the Western blots from three repeat experiments with the Q115L clone and measured the pS65-Ub levels with a previously developed sandwich ELISA [22], which corroborated a consistent and highly significant 25% reduction of pS65-Ub (Figure 7A), similar to what we had observed in the HEK293 overexpression paradigm.

For PINK1 D525N, we observed a strong reduction in endogenous PINK1 protein levels that was accompanied by a significant decrease in pS65-Ub levels. This effect was observed in each of the three independent clones that were used for experiments and quantified (Figure 7B). PINK1 and pS65-Ub levels were reduced by 75% and 50%, respectively, while PRKN levels remained unchanged. This dramatic loss of PINK1 stability and extent of pS65-Ub reduction had not been observed in D525N overexpressing HEK293 cells, suggesting that endogenous analysis is able to uncover additional defects of *PINK1* SNVs. Together, our analysis of gene-edited cells confirms a substantial loss of function for both the common PINK1 Q115L and the rare D525N variant in disease-relevant neuronal cultures. Additional studies are now warranted to determine their contribution to disease risk, age at onset, and clinical outcomes at the individual carrier level.

## 4. Discussion

Here, we characterized the effect of 20 SNVs in *PINK1* and *PRKN* utilizing a series of functional analyses in human cells. Most of the common variants, as to be expected by their higher frequency, did not show large functional defects. In fact, a discernible loss of activity was only observed for the common substitution PINK1 Q115L. While also some of the rare mutations (PINK1 R207Q, C575R and PRKN A82E, T83A) did not show defects, almost half displayed clear loss-of-function phenotypes (PINK1 M318L, V418M, R501Q, and PRKN S65N, R275W, R275Q, P437L). Another smaller group exhibited an intermediate phenotype with reduced functional activity (PINK1 A339T, D525N and PRKN P343L). Overall, there was a significant negative correlation of functional activity and CADD scores, supporting the general value of the tool to predict the deleteriousness of variants. Of the SNVs with scores smaller than 20, some showed trends of reduced activity, however statistically they all behaved similar compared to their cognate WT enzyme. Nevertheless, variants with CADD scores between 20 and 30 were functionally quite divergent and such should be further characterized in functional studies like ours.

Our results for the PINK1 and PRKN substitutions are in good agreement with other comprehensive studies that detailed genetic variants in *PINK1* [40] or *PRKN* [32]. Consistent with our data, the common PRKN substitutions A82E, S167N, V380L, and D394N showed no effect in mitophagy assays, while expression of the rare R275W and P437L mutations resulted in significant reduction of mitophagy compared to PRKN WT [32]. In addition, our analysis for S65N concurs with data for the non-phosphorylatable PRKN S65A mutant, which had been generated in cell and mouse models to investigate the role of the PINK1-dependent phosphorylation at this site. The results for both mutants are consistent with an important priming role required for full activation of PRKN [31,41]. Consistently, skin fibroblasts from patients with the rare PRKN S65N mutation also showed reduced substrate ubiquitylation and lower pS65-Ub levels [31]. As for PINK1, and in line with our results, PINK1 Q115L, commonly classified as benign, consistently resulted in a moderate loss of function compared to WT [40]. The two other common variants, PINK1 A340T and N521T, also showed some but less pronounced reduction in activity compared to WT in the same study [40]. However, while the PINK1 R501Q variant with a CADD score of 32 failed to stabilize upon CCCP in our hands, it was reported to function like WT in another study [35]. Disparities in the functional scores between studies might be caused by differences in the experimental approach such as variations in expression levels, time points, and readouts. Due to the continuous import and cleavage of PINK1 in healthy mitochondria, endogenous PINK1 is barely detectable in unstressed cells but swiftly accumulates upon depolarization of mitochondria. Even with very low exogenous expression, as used here for the first part of the study, stabilization of PINK1 is challenging to observe because of the continuous supply of PINK1. Consistently, we previously uncovered a destabilizing effect of the PINK1 I368N mutation that was only detectable endogenously, in patient skin fibroblasts, but not upon overexpression of this mutant [42]. Likewise, when testing the effect of PINK1 D525N endogenously in neurons, we discovered that it resulted in significantly lower PINK1 protein levels in all three independent clones analyzed. This effect was not apparent upon transient expression of this mutant, confirming that overexpression of PINK1 is prone to mask such cryptic phenotypes. Interestingly, both Q115L and D525N are located just outside two α-helical regions in PINK1 that were recently shown to interact with each other and to be important for PINK1 activation in cells [43]. Several other changes in these regions presented additionally with reduced auto- and substrate phosphorylation, suggesting that intramolecular interaction between an N- and C-terminus of PINK1 are critical for its stabilization at the TOM complex [43].

One advantage of gene-editing over other methods for stable low expression of genes, are that it results in cell-type dependent expression levels and those might be important to fully appreciate the impact of a mutation in a certain cellular context. PRKN levels for example, are quite low in non-neuronal cells such as undifferentiated ES or neuronal precursor cells but highly upregulated in neurons (Fiesel et al., submitted and [44]). A clear limitation of our and other similar studies is the use of CCCP as non-physiological, acute mitochondrial damage inducer. While this paradigm is convenient and results in reliable activity readouts, alternative stressors such as Oligomycin/Antimycin have been shown to have fewer effects on non-mitochondrial organelles [45]. In addition, the field should strive to integrate more subtle, chronic conditions to study potential long-term mitophagy defects. Such studies could also determine potential effects on cell viability of a certain variants such as PINK1 D525N upon mild mitochondrial stress or treatment with PD related neurotoxins, such as rotenone or paraquat. After all, PD is an age-related disorder, and it is conceivable that complete loss of mitophagy variants in PINK1 and PRKN leads to early onset, while variants with subtle defects would increase the risk for late onset disease due to the accumulation of defects over decades. The same rationale speaks for a disease predisposition caused by heterozygous mutations, which has been discussed for the past 15 years and is still debated [46,47,48,49,50,51,52,53]. Using CRISPR/Cas9 in meaningful cell models, the effect size of certain heterozygous mutations in PINK1 and PRKN can now be revisited. In addition, gene-editing will be fundamental to study multi-genic impacts of combination of variants with small effect size in one or several pathway genes such as rare occurrences of di-genic mutations in PINK1 and PRKN [53,54,55]. Finally, while numerous studies have categorized non-synonymous mutations functionally, CRISPR/Cas9 paired with sensitive readouts also opens new avenues to study potential functional deficits of synonymous or non-coding variants in *PINK1*, *PRKN*, or other mitophagy genes to determine their pathogenic role for PD.

## Figures and Tables

**Table 1:** Common and rare variants in *PRKN*. Common (>1%) and rare variants (<1%) in PRKN are listed together with their color-coded domain, the caused amino acid change and the allele frequencies (AF) in GnomAD, the PPMI cohort and a familial PD cohort. Shown is also the CADD score, a computational prediction for the deleteriousness of the mutation. CADD scores were generated with GRCh38 v1.5.

	Affected Domain	aa Change	GnomAD AF [%]	PPMI AF [%]	Familial PD AF [%]	CADD Score
Common	R0/UPD	S167N	6.849	2.691	2.747	14.71
	REP	V380L	16.430	16.760	16.044	2.42
	REP	D394N	2.540	3.868	5.385	22.9
Rare	UBL	S65N	0.001	0.112	0.000	24.40
	Linker	A82E	0.353	0.336	0.440	2.20
	Linker	T83A	0.009	0.000	0.110	0.23
	R1	R275W	0.194	0.336	0.440	23.90
	R1	R275Q	0.002	0.000	0.000	26.80
	IBR	P343L	0.008	0.056	0.000	26.90
	R2	P437L	0.156	0.280	0.330	25.00

**Table 2:** Common and rare variants in *PINK1*. Common (>1%) and rare variants (<1%) in PINK1 are listed together with their color-coded domain, the caused amino acid change and the allele frequencies (AF) in GnomAD, the PPMI cohort and a familial PD cohort. Shown is also the CADD score, a computational prediction for the deleteriousness of the mutation. CADD scores were generated with GRCh38 v1.5.

	Affected Domain	aa Change	GnomAD AF [%]	PPMI AF [%]	Familial PD AF [%]	CADD Score
Common	NT	Q115L	3.363	4.036	7.033	21.00
	Kinase C-lobe	A340T	8.821	5.213	4.396	5.12
	CTD	N521T	29.240	26.682	25.055	17.3
Rare	Kinase N-lobe	R207Q	0.016	0.000	0.000	0.07
	Kinase N-lobe	M318L	0.090	0.056	0.110	24.50
	Kinase C-lobe	A339T	0.057	0.168	0.659	23.70
	Kinase C-lobe	V418M	0.001	0.056	0.000	31.00
	Kinase C-lobe	R501Q	0.323	0.056	0.000	34.00
	CTD	D525N	0.007	0.056	0.000	23.70
	CTD	C575R	0.000	0.000	0.000	15.85

## Data Availability

All data generated or analyzed during this study are included in this published article (and its supplementary information files).

## Supplementary Materials

The following supporting information can be downloaded at: https://www.mdpi.com/article/10.3390/cells11152426/s1, Figure S1: PINK1 WT but not PINK1 R501Q stabilizes after CCCP treatment.
