# Supplementary material for: Systematic Functional Analysis of PINK1 and PRKN Coding Variants
Source: Cells. 2022 Aug 5;11(15):2426. doi: 10.3390/cells11152426 (PMC9367835; doi:10.3390/cells11152426)

Supplementary Figure S1. PINK1 WT but not PINK1 R501Q stabilizes after CCCP treatment. (A) PINK1-V5/6xHis WT cDNA was transfected into HEK293E PINK1 KO using a previously optimized protocol [26], 24 h later cells were treated with either MG132 or CCCP for 4h. Western blots were probed with GAPDH and V5 antibodies to assess loading control and the levels of total PINK1 kinase, respectively. (**B**) HEK293E PINK1 KO cells were transfected with either PINK1-V5/6xHis WT, kinase-dead (KD) or the identified genetic PINK1 variants and treated with CCCP for 4h, as indicated. Western blots were probed with V5 antibodies to assess the levels of total PINK1 kinase. pS65-Ub antibodies were used to determine the amount of PINK1 product. VCL was used as loading control.

Supplementary Figure S1

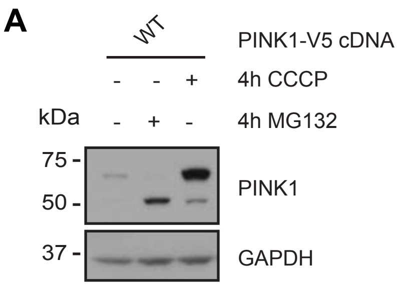

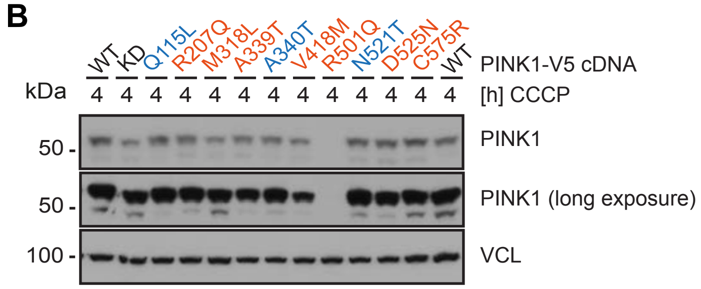

Supplement: Supplementary file 1 [file cells-11-02426-s001.zip › cells-1822522-supplementary.pdf]
